# Cytokinin derivatives as tools to enhance plant stress resilience

**DOI:** 10.3389/fpls.2026.1911890

**Published:** 2026-08-10

**Authors:** Jan F. Humplík, Thomas Schmülling, Martin Hönig

**Affiliations:** 1 Department of Chemical Biology, Faculty of Science, Palacký University, Olomouc, Czechia; 2 Institute of Biology/Applied Genetics, Dahlem Centre of Plant Sciences, Freie Universität Berlin, Berlin, Germany

**Keywords:** abiotic stress, biotic stress, cytokinin, cytokinin derivatives, cytokinin paradox, plant stress response

## Abstract

Cytokinins (CKs) are key regulators of plant growth and development. Recently, CKs have been increasingly recognized to have additional roles as modulators of plant adaptation to abiotic and biotic stresses. A large part of CK action in stress adaptation can be attributed to the promotion of antioxidative defense mechanisms. However, the contributions of CKs to stress tolerance can be complex and sometimes contradictory, as both increased and decreased CK signaling can enhance stress resilience depending on the context, a phenomenon referred to as the “cytokinin paradox”. In addition, some effects of exogenously applied classical CKs, including inhibition of root elongation and stimulation of stomatal opening, may become detrimental under severe stress. Recent research has therefore focused on developing advanced CK-derived compounds that selectively enhance stress tolerance but possess reduced classical growth-regulatory activities of CKs. These advanced derivatives, including *N9*-substituted CKs, CKX inhibitors, and urea-based compounds, retain antioxidant and photoprotective functions while minimizing developmental penalties. Their potential as tools to improve the stress resilience of crop plants is discussed.

## 1 Introduction

During recent years, the understanding of the biological functions of the CK class of plant hormones, which are chemically *N*^6^-substituted adenine derivatives, has been extended considerably. CKs were originally identified in the mid-20th century as substances essential to cytokinesis and, together with auxin, for shoot formation in cultured plant cells (Miller et al., 1956). Since then, numerous functions in regulating a diversity of plant developmental processes have been attributed to CKs. CKs have been shown to orchestrate a vast array of physiological processes, including shoot and root architecture, vascular development, chloroplast biogenesis, and the inhibition of leaf senescence (Werner and Schmülling, 2009; Kieber and Schaller, 2018). During recent years, it has become clear that CKs also play an important role in the plant´s response to abiotic and biotic stress (O’Brien and Benková, 2013; Pavlů et al., 2018; Cortleven et al., 2019). More broadly, CKs have essential functions in integrating environmental information in the endogenous developmental programs (Li et al., 2020).

In this review, we will shortly introduce the metabolism and signaling of CKs and give an overview of some well-documented activities in stress defense. More detailed information on the mechanisms of CK action in stress defense can be found in recent reviews (Bielach et al., 2017; Pavlů et al., 2018; Cortleven et al., 2019; Li et al., 2020; Savelieva et al., 2026). Thereafter, we will describe the attempts to synthesize compounds that retain the anti-stress activities of CKs but have lost the growth-regulating activities of endogenous and canonical synthetic CKs such as 6-benzylaminopurine (BAP) and kinetin. These two groups of CK (endogenous and canonical synthetic) induce similar biological responses, triggering the CK signaling pathway as described below.

### 1.1 Biosynthesis and metabolism of CK

The endogenous content of active CKs is recognized as highly dynamic, spatially heterogeneous, and subject to rapid fluctuations in response to developmental stages and external environmental cues (Zürcher and Müller, 2016). The maintenance of CK homeostasis is achieved through a tightly regulated balance of biosynthesis, irreversible degradation, reversible conjugation, and transport. The primary pathway for CK biosynthesis is initiated by the enzyme adenosine phosphate-isopentenyltransferase (IPT). IPT facilitates the rate-limiting step of transferring an isopentenyl moiety from dimethylallyl diphosphate (DMAPP) or hydroxymethylbutenyl diphosphate (HMBDP) to the *N^6^* position of adenosine phosphates (Kamada-Nobusada and Sakakibara, 2009). In higher plants, ADP/ATP are used as adenine source for IPT to synthesize isopentenyladenine (iP) and *trans-*zeatin (tZ) CKs, while the first step of *cis*-zeatin (cZ) CKs is catalyzed by tRNA-IPTs (Kasahara et al., 2004; Miyawaki et al., 2006). The initial products of the IPT reaction are CK nucleotides, which possess little or no biological activity. The conversion of these nucleotides into biologically active free bases is catalyzed by the LONELY GUY (LOG) family of phosphoribohydrolases. The *LOG* genes are ancient and widely distributed across bacteria, archaea, and eukaryotes, indicating that the ability to produce active CKs is a primitive trait (Kurakawa et al., 2007; Tokunaga et al., 2012). The irreversible degradation is catalyzed by CK oxidase/dehydrogenase (CKX) enzymes, which cleave the *N^6^* side chain from the adenine ring (Werner et al., 2006). The evolution of the *CKX* gene family appears to be an important adaptation for land plants. This indicates that as plants adapted to terrestrial environments, the requirements for a precise control of CK homeostasis to respond to a more variable and rapidly changing environment increased significantly (Wang et al., 2020). CK homeostasis is modulated not only by biosynthesis and degradation but also by the formation of CK conjugates, which can act as reversible storage forms or irreversible inactivation products. O-glucosylation of zeatin-type CKs generates conjugates that can be hydrolyzed back to active bases, providing a mechanism for transient buffering of CK levels (Brzobohatý et al., 1993; Hou et al., 2004). In contrast, *N7*- and *N9*-glucosides are considered metabolically irreversible, as no plant enzymes have been identified that efficiently reconvert them to free CKs, and these forms exhibit minimal biological activity (Mok and Mok, 2001). The accumulation of such irreversible conjugates can represent a major CK sink, contributing to long-term attenuation of CK signaling. Together, reversible and irreversible conjugation pathways provide plants with a flexible system to fine-tune CK availability and maintain hormonal balance.

### 1.2 CK signal perception and transduction

The perception and transduction of the CK signal occur through a multistep phosphorelay, an extended version of the ancestral two-component system found in prokaryotes (Hwang et al., 2002; Pekárová et al., 2016). The CK signal pathway comprises three protein types: sensor histidine kinases (HKs), authentic histidine phosphotransfer proteins (AHPs), and response regulators (RRs). The signaling cascade is initiated at the membrane of the endoplasmic reticulum and/or the plasma membrane by HK receptors. These receptors are hybrid kinases containing a sensory CHASE domain, a conserved HK domain, and a receiver domain. After the binding of a CK molecule to the CHASE domain, the receptor undergoes autophosphorylation at a conserved His residue. Subsequently, the phosphoryl group is transferred to an Asp residue within the receptor’s own receiver domain. The signal is then transmitted to the nucleus via AHPs, which act as redundant, positive regulators of the pathway (Müller and Sheen, 2007). CK binding by the receptor has been demonstrated to trigger the translocation of AHPs from the cytoplasm to the nucleus, where they transfer the phosphoryl group to nuclear-localized RRs (Hwang and Sheen, 2001). The RRs represent the final step in the phosphorelay and are categorized into three main types based on their structural domains and functional roles in gene regulation: B-type RRs (RRBs) function as transcription factors that directly activate the expression of primary response genes, whereas A-type RRs (RRAs) serve as CK-induced negative-feedback-loop regulators, and C-type RRs, which are not responsive to CK, nevertheless contribute to stress responses (Jeon et al., 2010; Kang et al., 2013).

### 1.3 CK functions in the response to abiotic stress

The role of CKs in the response to abiotic stress is multifaceted and depends on multiple factors. CK levels were observed to drop during abiotic stress, which was thought to allow for the dominance of stress-related hormones like abscisic acid (ABA) (O’Brien and Benková, 2013). Impaired CK signaling in plants with a reduced CK content or CK signaling has been shown to enhance resistance, particularly to drought (Nishiyama et al., 2011; Macková et al., 2013). However, recent work has indicated that increased CK signaling can also confer substantial benefits during stress responses, particularly by maintaining photosynthesis and reducing oxidative damage (Li et al., 2023).

#### 1.3.1 Drought and osmotic stress

Drought stress triggers a cascade of physiological changes aimed at conserving water, which are primarily regulated by ABA. In this context, CKs often act antagonistically to ABA, thereby modulating the extent and temporal dynamics of the response. While ABA induces stomatal closure and maintains root growth, exogenously applied CKs promote stomatal opening (Zeiger, 1983; Sharp and LeNoble, 2002). Although the stomata-opening role of CKs has been questioned—since reducing the CK content in *CKX* overexpressing plants does not always produce strong stomatal phenotypes—the beneficial effect of lowering endogenous CK levels on drought tolerance has been clearly demonstrated (Nishiyama et al., 2011). Consistently, endogenous CK concentrations typically decline in the xylem and leaves of drought-stressed plants due to reduced biosynthesis and enhanced degradation (Cortleven et al., 2019).

The final elements of CK signaling phosphorelay, the RRBs, negatively regulate drought stress responses in plants. Consequently, loss-of-function mutants of RRBs showed increased drought resistance in Arabidopsis (Nguyen et al., 2016). However, despite this negative role of CK signaling, an experimental increase of CK levels significantly enhanced plant drought tolerance (Rivero et al., 2010). Consistently, suppression of CKX activity improved water-use efficiency and the leaf net photosynthetic rate in drought-stressed cotton (Hu et al., 2025).

In addition, transgenic lines expressing an *IPT* gene under the control of a stress-inducible promoter exhibit a “stay-green” phenotype. These plants maintain higher photosynthetic rates and show a delayed leaf senescence under water-limiting conditions, resulting in improved grain yield (Rivero et al., 2010; Song et al., 2022). This is achieved through the redistribution of soluble sugars and the suppression of senescence-associated genes, helping the plant to maintain metabolic activity during the stressful period (Reguera et al., 2013). While constitutive expression of the CK degradation enzyme CKX resulted in limited shoot growth and permanent adaptation to drought, overexpression of the CK synthetic IPT enzyme caused significantly higher water loss. Conversely, the same IPT-overexpressing seedlings exhibited more rapid and significant recovery after rehydration (Prerostova et al., 2018). Although mutants impaired in their ability to perceive CK signals have repeatedly been reported to withstand drought stress better than wild type (Nishiyama et al., 2011; Zwack and Rashotte, 2015), artificially elevating CK levels has often produced similar effects. This contradiction in the proposed physiological role arises from the complexity of the CK response, which varies not only among organs but also with plant age and is also observed for the response to other types of abiotic stress (Cortleven et al., 2019).

##### 1.3.1.1 Paradox of CK signaling and metabolism in drought stress

The observation that enhanced plant resilience to drought can be achieved through both the upregulation and downregulation of CK signaling represents the so-called “cytokinin paradox” (Savelieva et al., 2026). A decrease in endogenous CK levels is considered a defensive strategy against severe water deficit, as reduced signaling promotes stomatal closure, prioritizes root system expansion over shoot growth to access deeper soil moisture, and triggers the recycling of nutrients for survival (Tran et al., 2007; Nishiyama et al., 2011; Mushtaq et al., 2022). Conversely, biotechnological strategies that increase the CK content—often employing stress-inducible promoters like *pSARK* to drive *IPT* gene expression—paradoxically improve tolerance by leveraging the hormone’s antioxidant properties to scavenge reactive oxygen species (ROS), inducing heat shock proteins (HSPs) to protect cellular structures, and delaying leaf senescence to maintain photosynthetic activity and grain yield under stress (Rivero et al., 2010; Peleg et al., 2011; Wang et al., 2020). This apparent contradiction is rationalized by the intensity and duration of the stress: while a radical decline in CK signaling is essential for survival during extreme, life-threatening drought, a moderate increase in CK signaling is more effective for maintaining productivity and cellular homeostasis during manageable, moderate stress episodes (reviewed by Savelieva et al., 2026).

#### 1.3.2 Salinity stress

Salinity is a severe abiotic constraint that limits crop productivity primarily by inducing osmotic stress and ion injury (Arif et al., 2020). In *Oryza sativa*, the RRB OsRR22 has been identified as a key negative regulator of salt tolerance (Takagi et al., 2015). Its loss-of-function leads to a dramatic increase in salinity tolerance (Zhang et al., 2019), as the *osrr22*, and even more the *osrr22;oshkt2* double mutant maintain a lower Na^+^/K^+^ ratio in their tissues by accumulating fewer sodium ions (Zhang et al., 2019; Liu et al., 2023). Similarly, the CK-signaling defective Arabidopsis triple mutants *ahp2,3,5*, and *arr1,10,12* showed almost 100% survival of severe salinity stress in comparison to only less than 5% of surviving WTs (Abdelrahman et al., 2021). Furthermore, cell-wall remodeling is promoted, and a robust antioxidant defense system is activated, including enzymes such as superoxide dismutase (SOD) and catalase (CAT), which protect cellular components from salt-induced oxidative damage (Nahar et al., 2022; Nongpiur et al., 2024; Aycan and Mitsui, 2026). However, in *Solanum lycopersicum* (tomato), salt treatment increased endogenous CK levels, supporting a role for CK in the salinity-defense mechanism (Keshishian et al., 2018).

#### 1.3.3 Temperature stress

The CK signaling pathway is intricately linked to the plants’ ability to withstand temperature fluctuations. In Arabidopsis, overexpression of *ARR7* resulted in increased sensitivity to freezing, whereas the *arr7* mutant exhibited enhanced tolerance to freezing (Jeon et al., 2010; Jeon and Kim, 2012). Under heat stress, CKs promote thermotolerance by upregulating the transcription of genes encoding heat shock proteins (HSPs). In this process, CKs collaborate with gibberellin (GA) and ethylene to regulate *HSP* expression via distinct signaling pathways (Zhang et al., 2025). CKs specifically protect the photosynthetic apparatus, particularly photosystem II (PSII), from heat-induced damage (Skalák et al., 2016). In addition, CK induces the production of small HSPs, which function as molecular chaperones, thereby preventing protein denaturation and assisting in the refolding of misfolded proteins under high-temperature conditions (Skalák et al., 2016; Zhang et al., 2025).

#### 1.3.4 Light stress and oxidative degradation

A key role of CKs is to protect the photosynthetic machinery from photoinhibition and oxidative degradation. Stress conditions, such as high light (HL) intensity or drought, typically result in the overproduction of ROS, including singlet oxygen and hydrogen peroxide. HL stress can inactivate PSII, with the D1 core protein being a primary target of damage (Cortleven et al., 2014). Under normal conditions, plants replace damaged D1 proteins through *de novo* synthesis and a repair cycle. CKs support this process by promoting the expression of the *psbA* gene, which encodes the D1 protein, and by maintaining steady-state D1 levels. In plants with a reduced CK status, D1 protein levels decrease significantly under HL stress, resulting in slow and incomplete recovery from photoinhibition. This protective function is facilitated by the AHK2 and AHK3 receptors, as well as the RRBs ARR1 and ARR12 (Cortleven et al., 2014). In addition, CKs bolster the plant’s antioxidant capacity through both enzymatic and non-enzymatic systems. They promote the activity of key ROS-scavenging enzymes, such as superoxide dismutase (SOD), ascorbate peroxidase (APX), and catalase. Furthermore, the levels of non-enzymatic antioxidants, including carotenoids, xanthophylls (e.g., neoxanthin), and tocopherols (e.g., α-tocopherol), are influenced by the CK status. These compounds are crucial for dissipating excess excitation energy and protecting membrane lipids from peroxidation. By maintaining these systems, CKs prevent the inhibition of the D1 repair cycle caused by excess ROS, thereby safeguarding the plant’s photosynthetic efficiency under adverse conditions (Cortleven et al., 2014, Cortleven et al., 2019). Conversely, the CK paradox (see 1.3.1.1) is also seen in the production of secondary metabolites. Whereas exogenous application of CKs such as BAP or kinetin led to an increase of phenolic compounds and flavonoids (Aremu et al., 2013; Ahanger et al., 2018), the absence of CK signaling in Arabidopsis mutants caused an elevated content of primary and also of secondary metabolites (flavonoids and sterols) as well (Abdelrahman et al., 2021).

### 1.4 CK functions in response to biotic stress

In addition to their well-characterized roles in abiotic stress regulation, CKs have also been identified as regulators of biotic stress. In *Arabidopsis thaliana*, CKs primarily enhance disease resistance through salicylic acid (SA)-dependent signaling. Choi et al. (2010) demonstrated that the CK-activated transcription factor ARR2 interacts with the SA response factor TGA3 in an NPR1-dependent manner, thereby inducing the defense gene *PR1* and the SA biosynthesis gene *ICS1*, and thus promoting resistance to *Pseudomonas syringae*. Further, treatment with CKs induces SA-dependent defense responses and enhances resistance to bacterial and oomycete pathogens. However, this effect is abolished in *npr1* and SA-deficient (*eds16*) mutants, which highlights the essential role of SA signaling (Choi et al., 2010; Argueso et al., 2012; Hönig et al., 2026). Most recently, CK (*t*Z) was shown to induce resistance that is dependent on both the CK receptor AHK3 and the SA-signaling regulator NPR1 (Hönig et al., 2026). A similar SA dependency of CK action has been observed in tomato (Gupta et al., 2021). In contrast, Johnston et al. (2026) reported that SA suppresses CK signaling, causing yield penalties. The detrimental effects of SA-based autoimmunity were overcome by an increase in CK signaling. This indicated that the relation between SA and CK in stress defense is complex and that CK functions in defense activation and growth maintenance need to be balanced (Johnston et al., 2026). Furthermore, in tobacco, CK-induced resistance has been shown to correlate with increased accumulation of antimicrobial phytoalexins, suggesting a distinct mechanism from that observed in Arabidopsis (Großkinsky et al., 2011).

## 2 The role of CK in plant redox regulation

The positive effect of CKs during stress adaptation could, in large part, be credited to the mitigation of ROS via enhancing plant antioxidative mechanisms (Hönig et al., 2018a). During severe stress, ROS are produced by plant metabolism, which arises from disruptions in cellular activities within organelles like the chloroplast, mitochondria, and peroxisomes (Choudhury et al., 2017). The formation of ROS is not only harmful, but it may also be beneficial to plant defense. While formerly ROS were only considered to be harmful agents that had to be eliminated, this view has changed. ROS, along with nitric oxide (NO), are recognized as essential signaling molecules that regulate developmental trajectories and environmental adaptations (Tognetti et al., 2017). For example, the maintenance of the shoot and root apical meristems is fundamentally dependent on precisely controlled spatiotemporal ROS gradients (Yang et al., 2018). The regulatory ROS bursts in meristems are precisely spatially regulated and consist of short-time emissions of free radicals that do not pose danger to developing organs (Zeng et al., 2017). Understanding ROS activities in meristems is complicated by the fact that different ROS may play different roles in meristem differentiation. Whereas superoxide (O_2_^−^) anion is abundant in stem cells and required to maintain their multipotency, hydrogen peroxide (H_2_O_2_) promotes cell differentiation in the peripheral meristematic zone. The balance between O_2_^−^ and H_2_O_2_ controls plant stem cell fate by antagonistically regulating the expression of *WUSCHEL*, which is required for shoot meristem proliferation and maintenance (Zeng et al., 2017). The intricate regulatory network linking CK-responsive factors, ROS, and core meristem regulators—including WUSCHEL, SHOOT MERISTEMLESS, and CLAVATA proteins—should be considered when applying exogenous CKs to modulate stress responses (Jayaraman et al., 2015; Tognetti et al., 2017; Porcher et al., 2021).

During abiotic stresses, the uncontrolled production of ROS often exceeds the plant’s buffering capacity, leading to oxidative damage to lipids, proteins, and the photosynthetic apparatus (Bielach et al., 2017). Plants are equipped with a system of reductases and antioxidants to reduce the harmful effects of ROS (Gill et al., 2013), and CKs significantly upregulate both enzymatic and non-enzymatic antioxidant mechanisms (Hönig et al., 2018a). Exogenous application or endogenous elevation of CKs has been shown to enhance the activities of key enzymes, including superoxide dismutase (SOD), which constitutes the first line of defense by dismutating O_2_^−^ to H_2_O_2_, as well as catalase (CAT), ascorbate peroxidase (APX), and guaiacol peroxidase (POD), which facilitate the subsequent breakdown of H_2_O_2_ into water and oxygen (Zavaleta-Mancera et al., 2007; Chang et al., 2016; Xu et al., 2016). Taken together, CKs have an important role in protecting plants against ROS. However, the same mechanisms that are beneficial in stress protection may also negatively affect meristem function and thus developmental processes.

## 3 Limitations of canonical CKs as anti-stress compounds

Soon after their discovery, CKs were considered as potential agrochemicals that may help plants cope with unfavorable environments (Koprna et al., 2016). CKs, mostly BAP and kinetin, were successfully tested in numerous studies in various crops, including cereals, legumes, vegetables, and fruit trees, to improve different traits from grain yield to fruit size (Hussain et al., 2025; Koprna et al., 2016). Regarding stress mitigation, they have been successfully used against abiotic stresses, particularly salt stress, to improve grain yield under these conditions. However, despite extensive testing, they haven’t found a stable place among the commercialized plant growth regulators, mainly due to the complexity of their effects (Koprna et al., 2016).

The term “canonical CK” refers primarily to their ability to trigger CK responses through the canonical two-component signaling pathway (Hwang et al., 2002) but it includes as well a well-known set of biological downstream activities. Therefore, we can define a canonical CK as a compound (naturally occurring or synthetic one) that activates the CK receptor pathway, stimulates shoot growth, reduces root elongation, counteracts ABA in stomata regulation, and protects chlorophyll and photosystem against degradation during wilting or senescence. Under optimal growth conditions or under mild stress, plants will benefit from most of these CK effects, however under severe stress most CK activities tend to worsen the plants´ conditions (Savelieva et al., 2026). For example, under drought stress, treatment with canonical CKs (tZ, BAP, or kinetin) will probably not improve the situation of the struggling plant. Depending on the concentration used, it may stop root growth (Stenlid, 1982), causing an even higher need for water. Moreover, the stomata remain open when exogenous CK suppresses the natural ABA effect (Farber et al., 2016), and, finally, the production of the pro-senescent hormone ethylene may be stimulated in certain cases (Chae et al., 2003; Yamoune et al., 2024). A possible way to overcome these limitations lies in the structure-activity relationship (SAR)-guided synthesis of novel CK-derived compounds that are modified to avoid negative effects (Podlešáková et al., 2012), while retaining a strong capacity to induce anti-oxidative mechanisms. In our view, the ability to improve the plant redox status may be considered as the most important property of these CKs and the basis of their stress alleviation activity. As described below, this effect is not dependent on other CK actions and thus can be separated from them.

**Figure 1:**
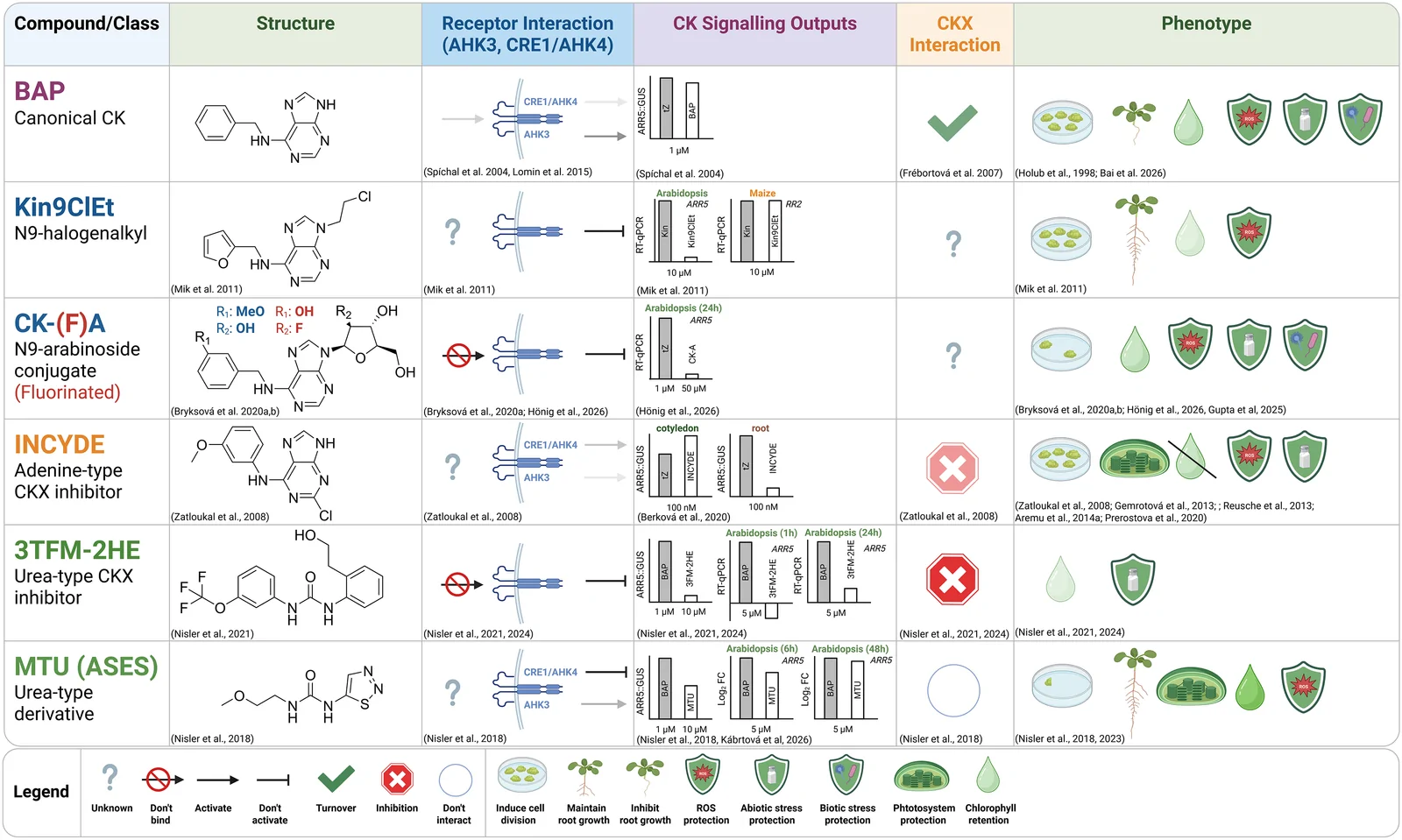
Advanced cytokinin derivatives and their activities in plant stress protection. The image compares compound structure, interaction with CK receptors (AHK3, CRE1/AHK4), effects on canonical CK signaling (measured via plant reporter construct *ARR5::GUS* and/or RT-qPCR/RNAseq outputs), CKX interaction, and major physiological outcomes, including callus proliferation, maintenance of root growth, oxidative stress protection, abiotic and biotic stress tolerance, photosystem stabilization, and chlorophyll retention. When the corresponding phenotype symbol is missing, the activity was not published. A crossed symbol indicates a lack of activity. The intensity of the symbol represents relative activity, where comparable. The number of calluses on the Petri dish indicates relative activity in the tobacco callus bioassay. BAP, 6-benzylaminopurin; Kin9ClEt, 6-furfurylamino-9-(2-chloroethyl)purine; CK-A, cytokinin arabinoside; CK-FA, fluorinated cytokinin arabinoside; INCYDE, 2-chloro-6-(3-methoxyphenyl)aminopurine; 3TFM-2HE, 1-(2-(2-hydroxyethyl)phenyl)-3-(3-(trifluoromethoxy)phenyl)urea; MTU, (1-(2- methoxyethyl)-3-(1,2,3-thiadiazol-5yl)urea).

**Figure 2:**
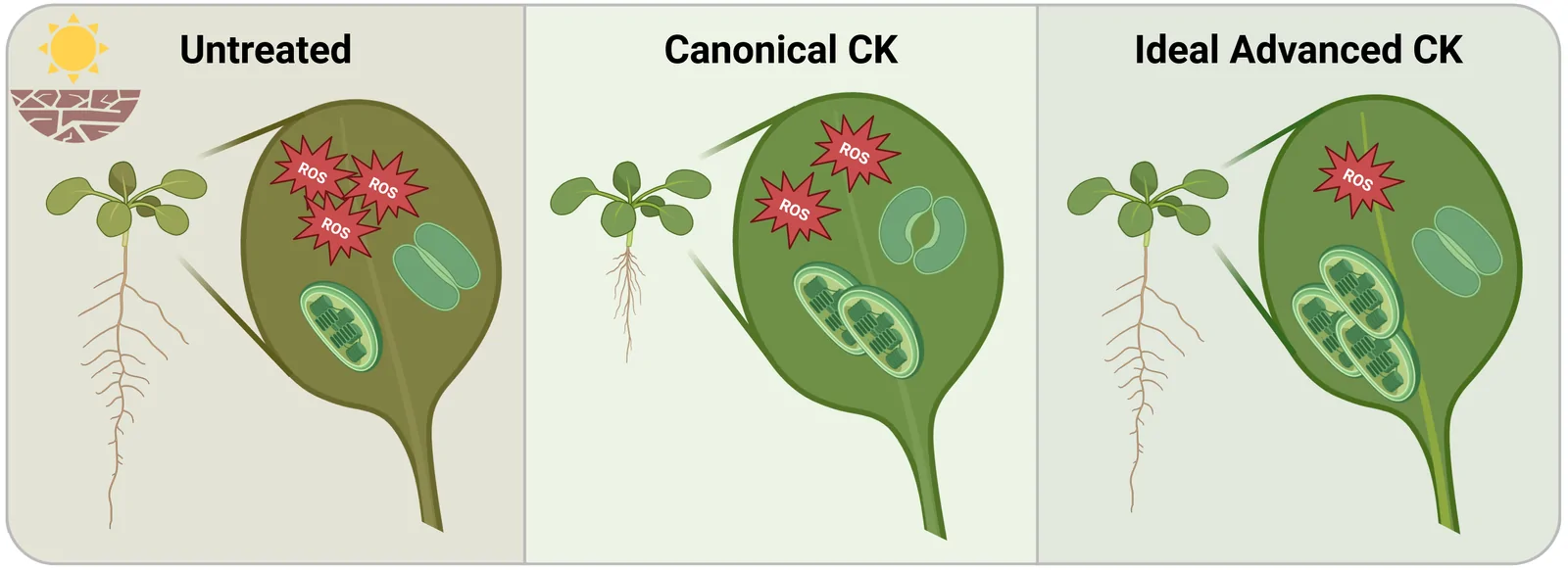
A comparison of the physiological responses of a plant under drought stress to a canonical CK and an ‘ideal’ advanced CK derivative. In the control plants, high levels of ROS damage, chlorophyll loss, and a reduced photosynthetic ability are typically observed, yet root development proceeds normally, and stomata close in response to insufficient water potential. In plants treated with a canonical CK, a lower level of ROS is produced, chlorophyll and photosystem activity are protected, but root growth is strongly inhibited and stomata remain open long after the first signals of drought. Plants treated with an ideal modern CK derivative maintain chlorophyll and photosystem activity, suppress excessive ROS accumulation, preserve root growth, and immediately close stomata upon the first signal of harmful water loss.

In the next sections, we will review the state of the art of the synthesis and testing of advanced CK-derivatives (**Figure 1**) lacking detrimental activities on plant growth (**Figure 2**). They are structurally related to canonical CKs but may even lack the ability to activate CK receptors, but importantly, they retain the ability to mitigate the consequences of stress.

## 4 Advanced CK derivatives in plant stress protection

**Figure 3:**
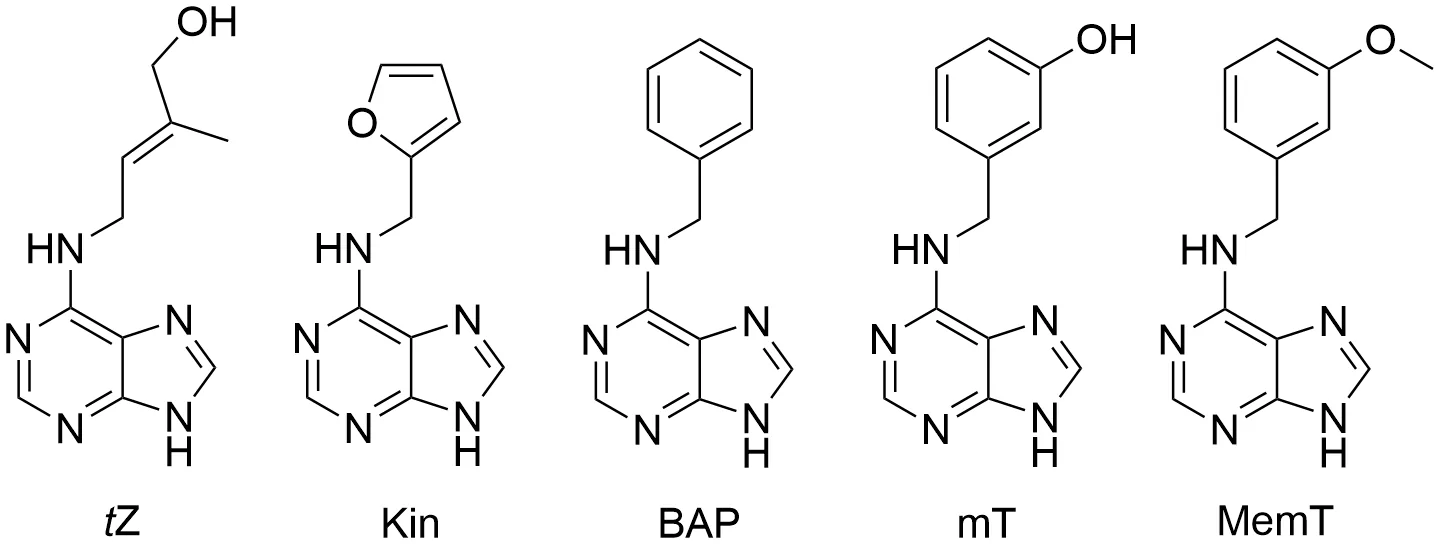
Structures of CK bases. tZ, trans-zeatin; Kin, kinetin; BAP, 6-benzylaminopurin; mT, meta-topolin; MemT, meta-methoxytopolin.

The first structural motif that was described to mitigate some of the negative effects of exogenously applied canonical CKs was found in nature, specifically in poplar leaves (Strnad, 1997). Topolins (T) (**Figure 3**), endogenous aromatic hydroxy CK derivatives of BAP, were discovered in the late 1990s and have since become a valuable alternative to BAP in micropropagation systems. The presence of a hydroxy or a methoxy group on the benzene ring of T enables the formation of O-glucosides, a reversible storage form that prolongs CK activity. In addition, the formation of the irreversible inactivation product, the 9-glucoside of meta-T (mT), is significantly reduced when compared with BAP (Bairu et al., 2011). Consequently, sufficient shoot production is achieved by T, accompanied by acceptable *in vitro* root formation and superior rooting during acclimatization when compared with BAP (Aremu et al., 2012a; Aremu et al., 2012b, Doležal and Bryksová, 2021).

**Figure 4:**
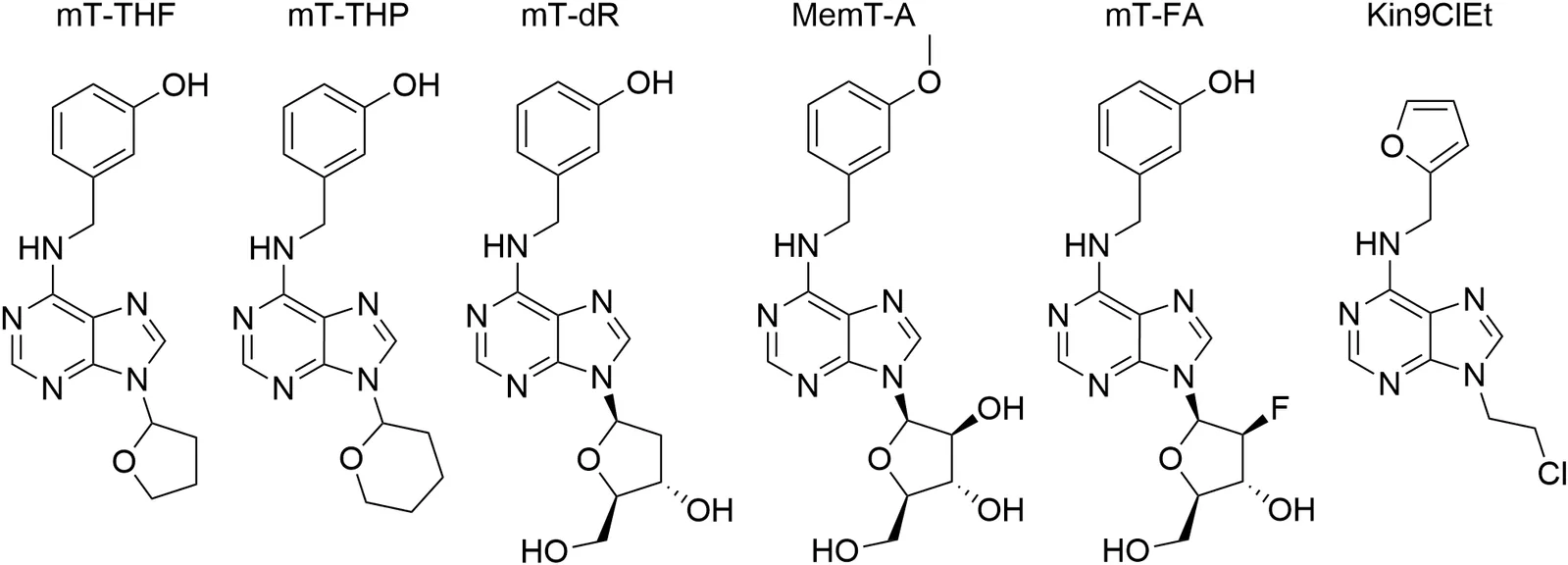
Structures of *N9*-substituted CK derivatives. mT-THF, 6-(3-hydroxybenzylamino)-9-tetrahydrofuran-2-ylpurine; mT-THP, 6-(3-hydroxybenzylamino)-9-tetrahydropyran-2-ylpurine; mT-dR, 6-(3-hydroxybenzylamino)purine-9-(2′-deoxy-β-D-ribofuranoside); MemT-A, 6-(3-methoxybenzylamino)purine-9-(β-D-arabinofuranoside); mT-FA, 6-(3-hydroxybenzylamino)purine-9-(2′-deoxy-2′-fluoro-β-D-arabinofuranoside); Kin9ClEt, 6-furfurylamino-9-(2-chloroethyl)purine.

Building on these findings, subsequent research diverged into two principal directions. One branch focused on derivatives that retain strong CK activity and are therefore primarily employed in plant biotechnology, particularly micropropagation. This includes T ribosides (Doležal et al., 2007), deoxyribosides (represented by meta-topolin deoxyriboside, mT-dR) (Matušková et al., 2020), and CK tetrahydropyranyl (THP) or tetrahydrofuranyl (THF) conjugates (represented by meta-topolin tetrahydrofuranyl conjugate, mT-THF, and meta-topolin tetrahydropyranyl conjugate, mT-THP) (Hönig et al., 2018a; Szüčová et al., 2009) (**Figure 4**). These compounds represent primarily stabilized or slowly releasable sources of active free bases and therefore preserve strong CK-dependent responses, including shoot induction and sustained meristematic activity, while mitigating the limitations of canonical CKs such as induction of shoot tip necroses, rooting inhibition and acclimatization failure. Consequently, their application has been directed mainly toward biotechnology and plant micropropagation (Doležal and Bryksová, 2021).

The second branch, which is the focus of this review, aims to develop CK derivatives that enhance the protective functions of CKs, such as stabilizing the photosynthetic apparatus and controlling oxidative stress. At the same time, these compounds should have minimal or no detrimental effects on root growth, stomatal regulation and growth–defense trade-offs. Over the past three decades, structure-activity-relationship studies (Doležal et al., 2007; Szüčová et al., 2009; Mik et al., 2011; Hönig et al., 2018b; Matušková et al., 2020) utilizing CK bioassays (Holub et al., 1998), receptor binding (Lomin et al., 2015), and receptor activation (Spíchal et al., 2004) assays together with CKX interaction assays (Kopečný et al., 2010) helped to identify several CK derivatives that at least partially uncouple stress protection activities from classical CK-driven growth responses.

### 4.1 *N9*-substituted CK derivatives

Several exogenously applied CK derivatives substituted at the *N9* position (**Figure 4**) have been shown to maintain or even to exhibit higher biological activity in comparison to the corresponding free base (Doležal et al., 2007; Szüčová et al., 2009; Mik et al., 2011; Hönig et al., 2018b; Matušková et al., 2020). These structural modifications can also mitigate the negative effects of treatment with canonical CKs (Aremu et al., 2017). This is possibly due to their reversible sequestration, which results in the sustained availability of active CK at physiological levels. Furthermore, specific *N9* substituents have been shown to increase resistance to enzymatic degradation by CKX. This metabolic stability, in turn, prolongs the effective half-life of both the parent compound and its metabolites (Podlešáková et al., 2012). Moreover, some *N9* substituents have been shown to decrease the accumulation of deactivation products, such as 9-glucosides (Aremu et al., 2017).

Additionally, THP substitution at the *N9*-position of the purine ring significantly enhanced acropetal transport of meta-methoxytopolin (MemT) and affected the distribution and levels of endogenous isoprenoid CKs across different plant tissues (Podlešáková et al., 2012).

Sugar or sugar-ring mimics, such as THF and THP, are among the most abundant substituents at the *N9* position of the purine scaffold, allowing a wide range of changes in biological activity (Vylíčilová et al., 2016). Recent studies have further highlighted the considerable potential of *N9*-substituted CK derivatives for enhancing plant tolerance to both biotic and abiotic stresses. The CK arabinose conjugate (CK-A) (**Figure 1**), more specifically, 6-(3-methoxybenzylamino)purine-9-(β-D-arabinofuranoside) (MemT-A), which was initially studied for its high antisenescent properties, was found to significantly suppress fungal infection in wheat and barley under field conditions (Bryksová et al., 2020a). Additionally, a strong synergy was observed upon co-treatment with the bacterial elicitor peptide flg22, leading to increased expression of *FRK1*, a downstream marker of the MAPK defense pathway (Bryksová et al., 2020a). Subsequent *in vivo* studies have shown that pretreatment with CK-A effectively induces resistance against *P. syringae* pv. tomato (*Pst*) in *A. thaliana* (Hönig et al., 2026).

Additionally, CK-As were also found to effectively induce immune responses and promote resistance to the fungal pathogen *Botrytis cinerea* in tomato (Gupta et al., 2025).

Transcriptomic analysis in *A. thaliana* suggested that CK-A activity is at least partially mediated by MAPK-dependent pattern-triggered immunity (PTI) and MAPK signaling (Bryksová et al., 2020b; Hönig et al., 2026) The SA and ethylene (ET) pathways are essential for achieving CK-A-induced resistance against both bacterial and fungal pathogens (Gupta et al., 2025; Hönig et al., 2026). In Bryksová et al. (2020a), the importance of the JA pathway was highlighted; however, this conclusion may have been influenced by the experimental setup, which used excised leaves.

*In vitro* bacterial CK receptor binding assays have demonstrated that CK-A does not directly bind to or activate the sensor histidine kinases AHK3 or CRE1/AHK4 (Bryksová et al., 2020a; Hönig et al., 2026). Similarly, transcriptomic and gene expression analyses have shown that CK-As fail to induce the expression of primary CK response genes, which are typically upregulated by canonical CKs such as *t*Z or BAP (Bryksová et al., 2020a; Gupta et al., 2025; Hönig et al., 2026). However, despite the absence of direct binding, induced resistance to *Pst* DC3000, which is triggered by CK-A treatment, has been shown to require the AHK3 receptor in Arabidopsis (Hönig et al., 2026). This finding suggests either a slow release of active free base or a role for AHK3 in maintaining a basal level of CK signaling (Hönig et al., 2026).

Bioisosteres of CK-A containing a fluorinated sugar moiety (CK-FA) (exemplified by meta-topolin fluroarabinoside, mT-FA) (**Figures 1**, **4**) have been reported to enhance biological activity and increase chemical and metabolic stability (Bryksová et al., 2020b). In addition, CK-FAs were reported to exhibit stronger anti-senescence properties than their non-fluorinated counterparts and to effectively reduce *Botrytis cinerea* infection in tomato (Gupta et al., 2025). Like CK-A, their fluorinated derivatives induced plant immunity without strongly activating canonical CK signaling or directly inhibiting fungal growth. However, although ET signaling was reported to be required for CK- and CK-A-mediated immunity against *B. cinerea* (Gupta et al., 2021, Gupta et al., 2025) CK-FAs were observed to promote disease resistance even in an ethylene-insensitive mutant (Gupta et al., 2025).

Fluorinated CK-As also alleviated the negative effects of salt and osmotic stress. In *A. thaliana*, fluorinated CK-As improved early seed establishment, chlorophyll retention, and nutrient status under both optimal and stressful conditions. The compound 6-(3-hydroxybenzylamino)purine-9-(2′-deoxy-2′-fluoro-β-D-arabinofuranoside) (mT-FA) was particularly effective in promoting growth under optimal conditions and alleviating stress under salt and osmotic stress in a concentration-dependent manner (Bryksová et al., 2020b).

In addition to BAP, another highly utilized CK, kinetin, has undergone several structural modifications to enhance its biological activity, both on the *N^6^* and *N9* positions of the kinetin structure (Mik et al., 2011; Hönig et al., 2018b). It was shown that leaves treated with a halogenalkyl derivative of kinetin, 6-furfurylamino-9-(2-chloroethyl)purine (Kin9ClEt) (**Figures 1**, **4**) accumulated nearly 10% less malondialdehyde (MDA), a marker of lipid peroxidation and senescence, than those treated with kinetin, consistent with its strong antisenescent activity. Although Kin9ClEt does not directly activate the CK receptors AHK3 or AHK4, it effectively promoted tobacco callus growth. Unlike kinetin, Kin9ClEt showed even at high concentrations no inhibitory effects on callus growth, which is likely due to a reduced formation of toxic metabolites during prolonged cultivation. Kin9ClEt also caused only a minimal inhibition of root growth in Arabidopsis and maize. Gene expression analyses revealed species-specific responses: Kin9ClEt failed to induce CK marker genes in Arabidopsis but showed CK activity comparable to or greater than kinetin in maize, presumably attributed to interspecies differences in free base release rates (Mik et al., 2011).

### 4.2 CKX inhibitors

Another way to avoid the negative effects associated with a high dose of exogenous CK is to apply CKX inhibitors. These compounds can be used to increase the endogenous CK levels and thus CK signaling. In addition, these compounds may or may not activate CK receptors themselves. There are two types of CKX inhibitors, adenine- and urea-type, which are described and discussed in the following.

#### 4.2.1 Adenine-type CKX inhibitors

**Figure 5:**
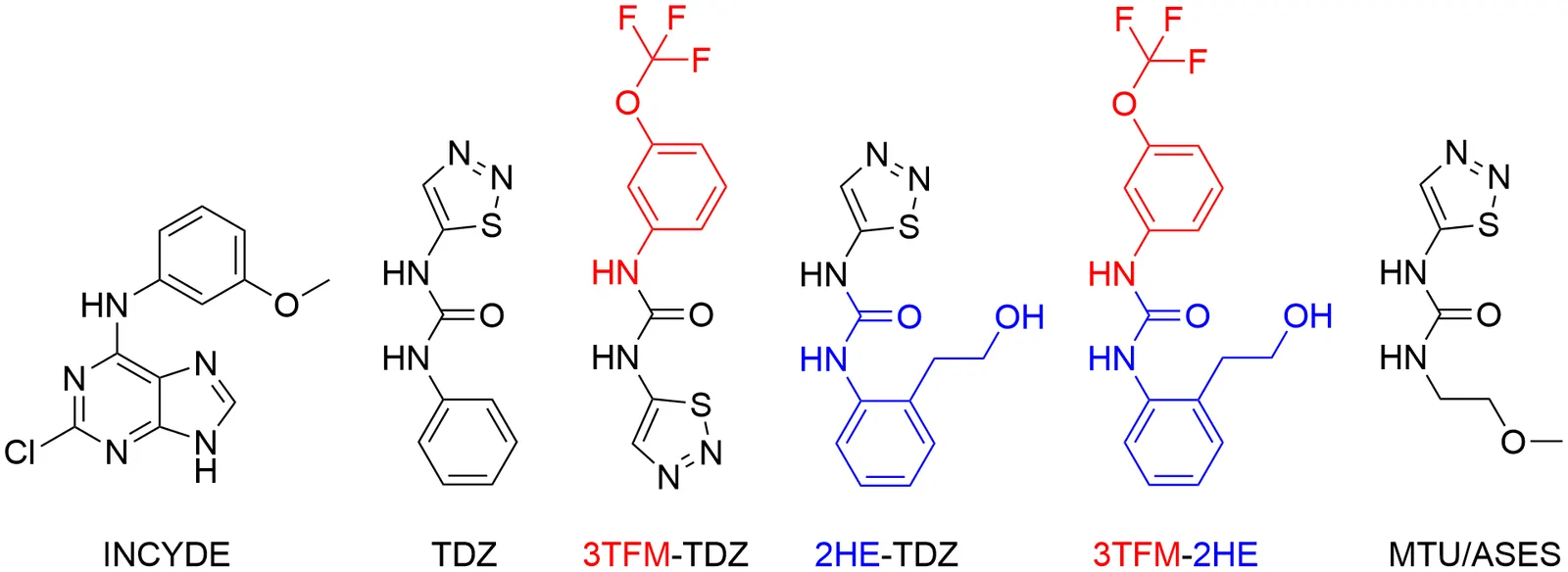
Structures of INCYDE and the urea-type CK TDZ and its derivatives. INCYDE, 2-chloro-6-(3-methoxyphenyl)aminopurine; TDZ, 1-phenyl-3-(1,2,3-thiadiazol-5-yl)urea; 3TFM-TDZ, 1-[1,2,3]thiadiazol-5-yl-3-(3-trifluoromethoxy-phenyl)urea; 2HE-TDZ, 1-[2-(2-hydroxyethyl)phenyl]-3-(1,2,3-thiadiazol-5-yl)urea; 3TFM-2HE, 1-(2-(2-hydroxyethyl)phenyl)-3-(3-(trifluoromethoxy)phenyl)urea; MTU/ASES, 1-(2-methoxyethyl)-3-(1,2,3-thiadiazol-5yl)urea. Red and blue colors show different structural motifs that were combined to design 3TFM-2HE.

Modifications of the *N^6^* substituent of the adenine moiety resulted in the identification of anilino purines, which are potent CKX inhibitors. The best studied example is INCYDE (2-chloro-6-(3-methoxyphenyl)aminopurine) (**Figures 1**, **5**). INCYDE exhibits high affinity for CKX but activates AHK receptors only to a limited extent (Zatloukal et al., 2008). The application of INCYDE has been shown to modulate endogenous CK homeostasis, thereby enhancing plant resilience to diverse environmental stressors (Gemrotová et al., 2013; Reusche et al., 2013; Aremu et al., 2014; Prerostova et al., 2020). In salt-stressed tomato plants, INCYDE application (10 nM) significantly mitigates oxidative damage by attenuating MDA accumulation and inducing robust antioxidant enzyme activities (SOD, CAT, and POD), while simultaneously preserving PSII efficiency and stimulating reproductive growth (Aremu et al., 2014). In a similar manner, INCYDE has been shown to reverse cadmium-induced growth retardation in medicinal species such as *Rumex crispus* and *Bulbine natalensis* at nanomolar concentrations, enhancing biomass and root architecture (Gemrotová et al., 2013). After infection by the vascular pathogen *Verticillium longisporum*, INCYDE has been demonstrated to stabilise CK levels, thereby delaying premature, pathogen-induced leaf senescence and, by extension, restricting fungal proliferation and the associated chlorotic symptoms (Reusche et al., 2013). It was determined that, at low nanomolar concentrations, the impact of INCYDE on growth and development of Arabidopsis seedling was not significantly different from that of *t*Z treatment. However, proteomic profiling in *Arabidopsis thaliana* revealed that INCYDE elicits a “stress response attenuation” phenotype characterized by the depletion of stress-associated factors, such as ABA signaling components and jasmonate biosynthetic enzymes, alongside the accumulation of proteins critical for ribosome biogenesis and chloroplast development (Berková et al., 2020). However, the efficacy of INCYDE in protecting from acute abiotic stressors, such as heat shock, depends on the timing of its application. While it mildly promotes recovery in acclimated plants, it may exacerbate negative stress effects in non-acclimated individuals, likely by delaying repair processes (Prerostova et al., 2020).

Application of INCYDE in field trials has yielded mixed results. The application of INCYDE in wheat and barley field trials did not enhance yields and did not change yield components (van Voorthuizen et al., 2021). In contrast, anisiflupurine, an analogue of INCYDE with a fluorine atom at the *C2* position of the purine moiety (INCYDE-F), has been successfully utilised to mitigate heat stress in paddy rice field trials (Leipner and Ruta, 2025). In flooded fields, its ability to open stomata was advantageous. Stomata opening induced by CKs leads to transpiration cooling and thus protects plants against heat stress. Applying anisiflupurine during the generative growth phase, before heat stress, secured seed setting and enhanced grain weight under heat stress conditions (Leipner and Ruta, 2025). These trials indicate that the advantageous effects of INCYDE/INCYDE-F may become apparent mostly in crop plants cultivated under stress conditions and present a promising approach to mitigating the negative consequences of stress. More work is needed to define the conditions, the dosage, and the timing of INCYDE/INCYDE-F application to obtain the best stress protection.

#### 4.2.2 Urea-type CKX inhibitors

In contrast to the high affinity to CKX and only a weak CK receptor activation shown by INCYDE (Zatloukal et al., 2008), the first generation of urea-based synthetic CK-like compounds, such as TDZ (1-phenyl-3-(1,2,3-thiadiazol-5-yl)urea) and CPPU (N-(2-chloro-4-pyridyl)-N’-phenylurea), bind to the CK-binding site of receptors and activate it strongly while simultaneously inhibiting CKX (Nisler, 2018). Thus, the long-lasting activity of TDZ is driven by its high receptor affinity, its CKX inhibitory profile, and the inability of CKX to degrade the ligand. These combined properties have led to the use of TDZ to promote fruit growth, improve the lifespan of cut flowers, enhance crop yield, and enhance stress tolerance (Nisler, 2018). In addition, TDZ has been shown to prolong the tolerance of potatoes (*Solanum tuberosum*) to early blight (*Alternaria solani*) and to exhibit a positive synergistic effect with certain fungicides in inhibiting necrosis progression (Pavlista, 2003). However, the strong receptor activation and long-lasting effects of TDZ are accompanied by pronounced inhibition of root growth and development, which are undesired effects (Nisler, 2018; Oshchepkov et al., 2023).

The synthesis of advanced urea-based (TDZ, CPPU) CK derivatives has thus aimed to generate compounds that exhibit only marginal or no intrinsic CK activity and display a very low affinity for CK receptors. These compounds have been shown to possess high CKX-inhibition activity, a property that was improved through studies of the CKX binding site structure (Kopečný et al., 2010; Nisler et al., 2016).

The discovery of the opposite orientation in the binding site of the CKX enzyme of the TDZ derivatives 3TFM-TDZ and 2HE-TDZ (Nisler et al., 2016) led to the design and the synthesis of the diphenylurea-derived compound 3TFM-2HE (Nisler et al., 2021) (**Figures 1**, **5**).

3TFM-2HE was reported to lack intrinsic CK activity, as evidenced by its total lack of affinity toward the AHK4 CK receptor, negligible activation of the CK reporter *ARR5:GUS*, and failure to independently induce shoot formation in tobacco or poplar tissue cultures (Nisler et al., 2021). Furthermore, this compound mitigated the growth penalties associated with salinity and osmotic stress by approximately 20% compared with stressed but untreated controls. Beyond the mitigation of stress, the promotion of growth has been observed in Arabidopsis, including an increase in leaf rosette size of up to 38% and an extension in plant lifespan of approximately ten days. The physiological benefits resulted in increased yield: Arabidopsis plants treated with 3TFM-2HE showed up to a 21% increase in total grain yield. Moreover, field trials on major crops demonstrated that 3TFM-2HE increases seed yield in spring barley, winter wheat, and winter rapeseed by up to 6.4%, 6.6%, and 7.5%, respectively (Nisler et al., 2021).

### 4.3 Other urea-type CK derivatives

Another derivative of the CKX inhibitor TDZ, MTU (also known as ASES) (**Figures 1**, **5**), has been shown to possess a remarkably elevated antisenescence activity (100-fold higher than BAP) in a wheat leaf senescence assay (Nisler et al., 2018). However, MTU/ASES does not appear to inhibit CKX activity. It was unsuccessful in activating the AHK4 receptor, while it could activate the AHK3 receptor to some extent, as well as the *ARR5:GUS* reporter gene construct (Nisler, 2018, Nisler et al., 2023). The most recent transcriptomic analysis demonstrated that MTU exhibits some but weaker CK activity than BAP at both 6 and 48 hours after treatment (Kábrtová et al., 2026). Furthermore, the existence of CK-independent response pathways has been proposed for this compound (Nisler, 2018).

MTU treatment has been shown to enhance the resilience of wheat plants to abiotic stresses, including drought, heat, and salinity. This enhanced resilience is characterised by several physiological responses, including faster recovery and higher survival rates following severe water deficit, maintained chlorophyll levels, a significant reduction in the accumulation of stress-induced ABA, lower ethylene production, and decreased oxidative damage. In addition, MTU does not inhibit root growth; rather, it has been observed to maintain and even stimulate root development in both wheat and Arabidopsis. Together, these activities resulted in a 5–8% increase in grain yields for field-grown wheat and barley (Nisler et al., 2023).

It has been shown that MTU significantly enhances the abundance of PSI-LHCa supercomplexes, effectively blocking their disintegration, which is the initial step of the leaf senescence process in cereals. This stabilization robustly promotes cyclic electron flow around PSI, providing a protective feedback loop that reduces excitation pressure on PSII and prevents stress-induced degradation of dimeric PSII and PSII-LHCII supercomplexes (Nisler et al., 2023).

Notably, recent studies have demonstrated that ethylenediurea (EDU) derivatives enhance drought tolerance in wheat, as evidenced by increased relative water content and improved root system architecture, including the stimulation of both primary and lateral root formation, an effect contrasting with that of TDZ (Oshchepkov et al., 2023, Oshchepkov et al., 2024). Although these phenotypic responses are interpreted by the authors as reflecting CK-like activity, direct experimental evidence supporting modulation of, or interaction with, canonical CK signaling pathways was not provided.

## 5 Concluding remarks

### 5.1 Common features of advanced cytokinins

Over the past two decades, substantial progress has been made towards the design of cytokinin-derived compounds to improve plant stress resilience while avoiding the detrimental side effects associated with canonical CKs. A comparison of the structurally diverse compounds reviewed here reveals a consistent functional pattern that may be regarded as a defining feature of advanced cytokinin derivatives.

Unlike canonical cytokinins, these compounds generally exhibit a pronounced reduction or complete loss of canonical CK activity, particularly of their ability to stimulate cell division and meristematic growth. This is most evident from their weak performance in standard bioassays, such as tobacco callus proliferation (Nisler, 2018, Nisler et al., 2021; Bryksová et al., 2020a, Bryksová et al., 2020b), their poor activation of CK receptors (Nisler et al., 2021, Nisler et al., 2023; Hönig et al., 2026), and their limited induction of primary CK response genes (Bryksová et al., 2020a; Nisler et al., 2021, Nisler et al., 2023; Hönig et al., 2026). From a physiological perspective, this attenuation of growth-promoting activity is highly advantageous under stress conditions, as it minimizes unwanted effects on root system architecture, stomatal behavior, and growth–defense trade-offs that often compromise stress tolerance when canonical CKs are applied exogenously (Koprna et al., 2016).

An important aspect is that the majority of these derivatives retain the capacity of CKs or even show an enhanced capacity to protect the photosynthetic apparatus (Doležal et al., 2007; Szüčová et al., 2009; Mik et al., 2011; Hönig et al., 2018b; Matušková et al., 2020; Nisler et al., 2021, Nisler et al., 2023) and regulate cellular redox homeostasis (Mik et al., 2011; Aremu et al., 2014, Aremu et al., 2015; Hönig et al., 2018a, Hönig et al., 2018b; Nisler et al., 2023). Independent of strong canonical CK signaling, these compounds consistently stabilize the chlorophyll content (Hönig et al., 2018b), preserve photosystem I (PSI) (Nisler et al., 2023) and PSII (Vylíčilová et al., 2016; Nisler et al., 2021) integrity, and reduce oxidative damage through enhanced antioxidant capacity (Hönig et al., 2018a). This combination of traits might be the main reason why treated plants better withstand abiotic and biotic stresses and recover faster after the stress is released.

The apparent functional uncoupling of CK growth promotion from stress-protective activity suggests that the latter represents a separable and targetable output of CK action. Importantly, although many of the described derivatives show little intrinsic CK activity, their biological effects can still depend on intact CK signaling components (Hönig et al., 2026), indicating that basal CK signaling competence may be required to unlock their protective potential. This concept is further supported by the activity of CKX inhibitors such as INCYDE, which enhance stress tolerance by stabilizing endogenous CK pools rather than directly activating CK receptors (Aremu et al., 2014). Notably, a recent study showed that the photoprotective effects of both BAP and MTU on the photosynthetic apparatus appeared to depend on CK and light-signaling pathway crosstalk (Kábrtová et al., 2026). Taken together, it suggests that these compounds do not simply bypass CK pathways but rather redirect CK outputs toward stress adaptation in an as yet unknown way.

Comparison of gene induction profiles of canonical CKs such as tZ, BAP, Kin, and even TDZ revealed that they indeed share a significant number of differentially expressed genes (DEGs), but each compound also activates specific pathways and functions (Großkinsky et al., 2011). It is therefore not surprising that a similar trend is observed when the gene expression profiles of advanced CK derivatives such as CK-A or MTU are compared to those of canonical CK bases (Hönig et al., 2026; Kábrtová et al., 2026). Despite the increasing number of gene expression studies of advanced CK derivatives (Bryksová et al., 2020a; Hönig et al., 2026; Kábrtová et al., 2026; Vylíčilová et al., 2016), a molecular marker that would indicate their specific activity profile is still to be identified. Further studies are needed to better understand the molecular mechanisms by which these CK derivatives retain or enhance the beneficial effects of CK under diverse stress conditions. Furthermore, their ability to prime the plants´ defense, as was shown for CKs (Choi et al., 2010; Großkinsky et al., 2011; Argueso et al., 2012), that is, to establish a state of increased readiness of treated plants to respond better to future stress (Hilker and Schmülling, 2019), should be investigated.

In the absence of molecular markers, current efforts to develop even more efficient CK derivatives still depend primarily on SAR studies built on the outcome of time-consuming bioassays, pot experiments, and field trials. Numerous SAR studies of adenine-type CK derivatives have been reported, focusing on modifications to the benzyl ring. As discussed above, the addition of hydroxy and methoxy groups to this ring provides numerous advantages, so, unsurprisingly, these are among the most successful structural modifications. Bioisosteres such as fluorinated compounds have also been shown to be highly effective in CK bioassays (Doležal et al., 2007). CK-As is only one of many groups of *N9*-CK conjugates (Vylíčilová et al., 2016). The most abundant of these are sugar conjugates, which provide potent candidates for advanced CK derivatives with a wide range of biological activities and are worth further testing.

A different approach can be seen for CKX inhibitors, especially urea-type, for which the molecular target is well-defined (Kopečný et al., 2010; Nisler et al., 2021). This has enabled the development of highly potent CKX inhibitors, which are among the most promising advanced CK derivatives with significant potential in stress biotechnology, as previously reviewed (Arora and Sen, 2022).

### 5.2 Advanced CKs in agriculture

From the perspective of agricultural application, environmental and mammalian safety represent important considerations in the development of advanced CK derivatives. Endogenous CKs and the synthetic CK Kin are classified by the U.S. Environmental Protection Agency (U.S. EPA) as substances with very low acute mammalian toxicity and are considered practically non-toxic, with no expected adverse effects on fish and wildlife (United States Environmental Protection Agency, 1995). Available data suggest that this favorable safety profile is largely retained by many advanced CK derivatives, including CK glucosides (Voller et al., 2010), THF and THP conjugates (Szüčová et al., 2009; Hönig et al., 2018b), arabinosides (Vylíčilová et al., 2016), deoxyribosides (Matušková et al., 2020), and urea-derived CKX inhibitors (Nisler et al., 2024), which generally exhibit low toxicity toward mammalian cells. An important exception is represented by CK ribosides, which exhibit pronounced cytotoxicity and have therefore attracted attention as potential anticancer agents (Voller et al., 2010). Their toxicity is associated with the ribose moiety, which enables intracellular conversion to the corresponding ribotides (Mlejnek and Doležel, 2005).

Thanks to advances in CK derivative research, we can finally see some new derivatives entering the agronomy market, in addition to commercial products like OnWard^®^ MAX containing classical kinetin. For example, IntraCrop utilises MTU in combination with pidolic acid and markets it under the name Status^®^. This product is said to protect the photosynthetic apparatus and improve plant resilience, resulting in quicker stress recovery and greater yield protection. Additionally, Chemap Agro’s AUCYT^®^ START utilises an unspecified CK derivative as biostimulant, which is designed to improve tillering. As mentioned above, Syngenta’s CKX inhibitor, anisoflupurine (INCYDE-F), was successfully tested in a series of field trials for protecting paddy rice from heat (Leipner and Ruta, 2025). However, the analogous compound, INCYDE, underperformed in cereal field trials, failing to enhance yields (van Voorthuizen et al., 2021). Only time will tell whether CK derivatives will establish themselves as effective and widely used compounds for the stress management of crops.
